# Using Linked Health Service Data in Multimodal Modeling of Kidney Transplant Waitlist Outcomes: Protocol for the Maximizing Organ Donor Utility Systemwide (MODUS) Study

**DOI:** 10.2196/67588

**Published:** 2025-07-29

**Authors:** Brenda Maria Rosales, Karan Shah, Nicole De La Mata, Heather Baldwin, James Hedley, Philip Clayton, Melanie Wyld, Patrick Kelly, Kate Wyburn, Rachael Morton, Angela Webster, MODUS Study Group

**Affiliations:** 1 Sydney School of Public Health Faculty of Medicine and Health University of Sydney Camperdown Australia; 2 NHMRC Clinical Trials Centre University of Sydney Sydney Australia; 3 Australian and New Zealand Dialysis and Transplant Registry Adelaide Australia; 4 Department of Renal and Transplant Medicine Westmead Hospital Sydney Australia; 5 Department of Renal Medicine and Transplantation Royal Prince Alfred Hospital Sydney Australia; 6 Central Clinical School Faculty of Medicine and Health University of Sydney Sydney Australia; 7 Westmead Applied Research Centre Westmead Hospital Westmead Australia; 8 see Authors' Contributions

**Keywords:** kidney failure, transplant waitlist, organ allocation, biovigilance, population study, health economic evaluation

## Abstract

**Background:**

Increasing deceased organ donation is a worldwide priority constrained by concerns of inadvertent transmission of cancer or infectious diseases from deceased organ donors. Up to 60% of potential donors referred for consideration for deceased organ donation in Australia do not proceed due to biovigilance concerns.

**Objective:**

We aim to describe the impact of accepting or declining potential donors foregone for biovigilance concerns on patient and transplant outcomes.

**Methods:**

The MODUS (Maximizing Organ Donor Utility Systemwide) study will use data for patients ever waitlisted for kidney transplantation and all potential donors referred for consideration for deceased organ donation. First, we will use binational data from the Australian and New Zealand Dialysis and Transplant Registry 2010-2020 to describe and evaluate factors impacting the current patient journey on the kidney transplant waitlist, including episodes of suspension and reactivation, time waiting, and whether transplanted. Second, we will quantify the time from offer decline to deceased donor transplantation and the impact of the intersectional disadvantage on the waiting time after decline for patients on the waitlist using flexible parametric survival models. Third, the MODUS study will use an established dataset of outcome data for all candidates for deceased organ donors referred to the New South Wales (NSW) Organ and Tissue Donation Service (OTDS) in 2010-2020 to describe donor referral risk profiles and determine any potential donor gains that could be made through better access to donor information at the time of decision-making, more accurate estimation of the absolute biovigilance risk, and varying of the acceptable biovigilance risk thresholds for accepting donors. Lastly, we will use the estimates derived from the first 3 aims as inputs for health economic models, where, using cohort- and individual patient-level simulations, we will quantify the impact of varying donor referral decisions on health care costs, quality-adjusted survival, the time on the waitlist, and the time to a kidney transplant.

**Results:**

Linked health data were received in 2023. Data analysis is ongoing, and results will be disseminated at scientific conferences, published in the scientific media, and published via collaborator networks in 2025.

**Conclusions:**

The MODUS study will provide evidence of the individual-level and health service effects of increasing acceptance of deceased donor kidneys that would otherwise be declined due to biovigilance concerns. Specifically, we expect to report our findings on improvements in overall patient survival and quality of life by increasing the number of waitlisted people transplanted from donors with an acceptable biovigilance risk who are currently foregone. We will also report on the cost-effectiveness of a potential “informed biovigilance strategy” versus current practice. In doing so, we will develop evidence to support policy and complex clinical decisions in Australia’s organ donor referral process with potential worldwide application.

**International Registered Report Identifier (IRRID):**

DERR1-10.2196/67588

## Introduction

Kidney failure is a leading cause of morbidity and mortality. Worldwide, the number of people on dialysis or with a kidney transplant has more than doubled since 1990 [1]. In 2010, over 4.9 million people worldwide had kidney failure, of which 2.6 million were on dialysis or had a kidney transplant [2]. Kidney transplantation is the preferred treatment for kidney failure as it increases patient survival, improves the quality of life, and is more cost-effective than dialysis, making it a compelling option for those who are suitable [3]. In Australia, kidney transplantation rates have steadily increased; despite this, demand continues to outweigh donor supply [4]. Increasing opportunities for kidney transplantation through deceased organ donation is, therefore, a worldwide and national priority [5].

To access a deceased donor kidney transplant in Australia, people on dialysis must be assessed as eligible for transplantation to enter the deceased donor waitlist [6]. Potential transplant recipients must understand and accept inherent risks from the procedure and ongoing treatment and have a high likelihood of significant benefits from transplantation to be eligible. Risk-benefit assessments are conducted by a multidisciplinary team that reviews clinical and surgical factors, mental health, social circumstances, and impacts. Any significant risk of death from comorbidity (eg, heart disease, cancer, infection) is a contraindication for transplant, and patients will not be eligible for the deceased donor waitlist. When a donor kidney becomes available, all waitlisted patients are considered by the national organ allocation algorithm, designed to find an immunologically compatible match, while also prioritizing those who have waited the longest or who are under 18 years. The algorithm stratifies matches by blood group and balances the time spent waiting with the degree of donor-recipient matching nationally and by state of donor origin to generate a list of potential donor-recipient pairs in ranked order. In Australia, the time spent waiting is measured from initiation of dialysis to account for delays in reaching the active waiting list due to medical issues or delayed transplant suitability assessment and to ensure the sense of fairness prioritized by consumers [7,8]. At the end of 2019, there were 1100 Australians on the deceased donor kidney transplant waitlist, with a median wait time of 1-4 years and a transplant rate of 7 per 100 years on dialysis [4].

Prior research has focused on factors impacting access to the waiting list or transplantation and subsequent posttransplant health outcomes [9-11]. However, the time spent waiting is underexplored. Kidney transplant waitlists are dynamic and change daily as new people are added and existing people are transplanted or become unwell and so are temporarily or permanently removed from the kidney transplant waitlist or die while waiting [12]. Patients can enter a variety of clinical states after being initiated onto the waitlist, impacting their experiences. Intercurrent illness, surgery, or investigations may necessitate temporary suspension or permanent removal from the waiting list. It has not been described how events occurring after waitlisting impact equitable access to transplantation.

**Figure 1:**
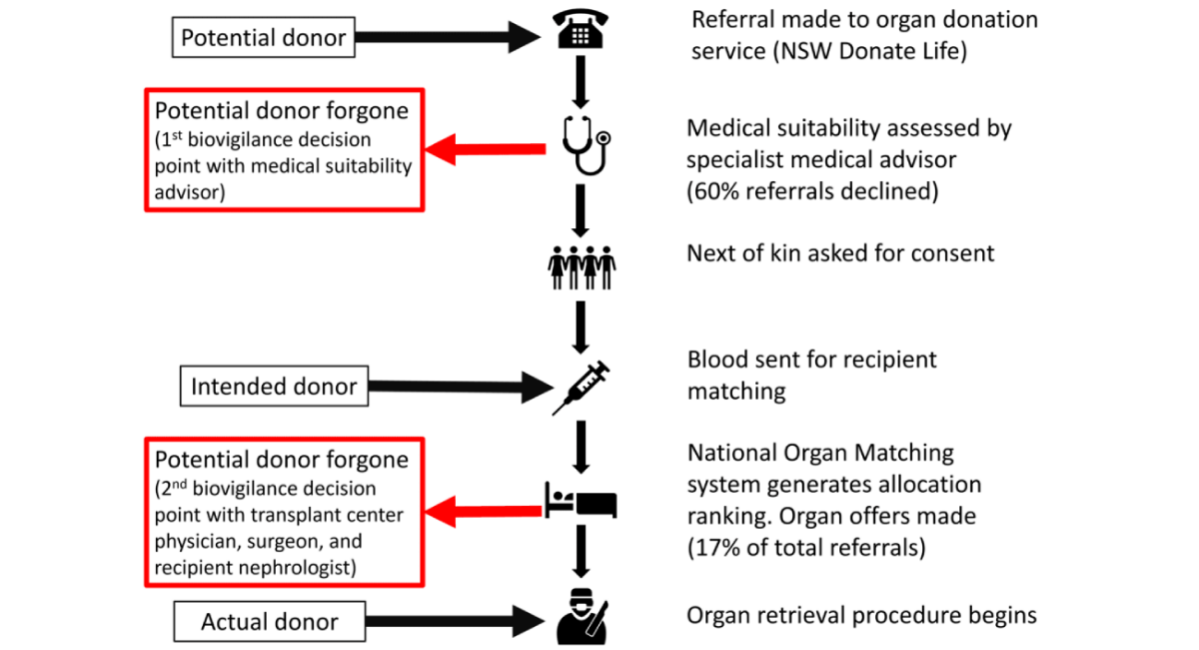
Schema of the NSW deceased organ donor referral process. NSW: New South Wales.

In 2009, the Australian government established a national program to increase capability and capacity within the health system to maximize organ donation rates [5]. Since 2009, the number of deceased organ donors has more than doubled in Australia [5]. Deceased organ donation is coordinated by the Organ and Tissue Authority, which has devolved to a national network of state-based donation specialist agencies called Organ and Tissue Donation Services (OTDSs). In Australia, New South Wales (NSW) has the greatest number of people waiting for a kidney transplant than other states and territories, as well as a more complex service delivery compared to other jurisdictions. Where some states have 1 kidney transplanting center, NSW has 8. Potential donors are identified by NSW hospitals and referred to the NSW OTDS for consideration for deceased organ donation (Figure 1) [13]. A donation specialist coordinates a thorough investigation, including medical history and assessment of the donors’ medical suitability for donation, while also seeking consent for a donation from their next of kin. Potential donors who meet the medical suitability criteria and have consenting next of kin (intended donors) enter the national allocation algorithm to be matched with kidney transplant candidates. Those who do not meet the medical suitability criteria or who were not accepted by transplant centers are considered potential donors foregone. Those who undergo surgery for organ retrieval are considered actual donors, even if their organs are not eventually transplanted.

**Figure 2:**
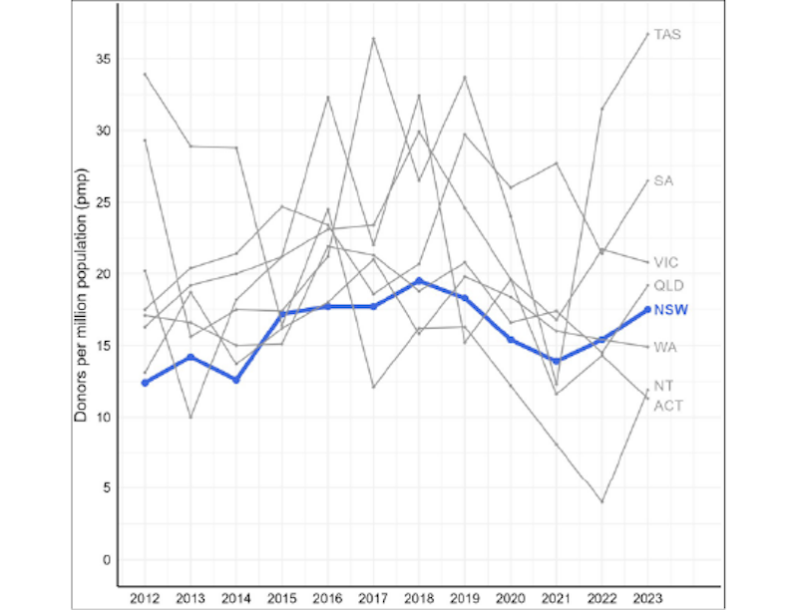
Deceased kidney donor activity by Australian state over time, per million population, 2012-2023. ACT: Australian Capital Territory; NSW: New South Wales; NT: Northern Territory; QLD: Queensland; SA: South Australia; TAS: Tasmania; VIC: Victoria; WA: Western Australia.

**Figure 3:**
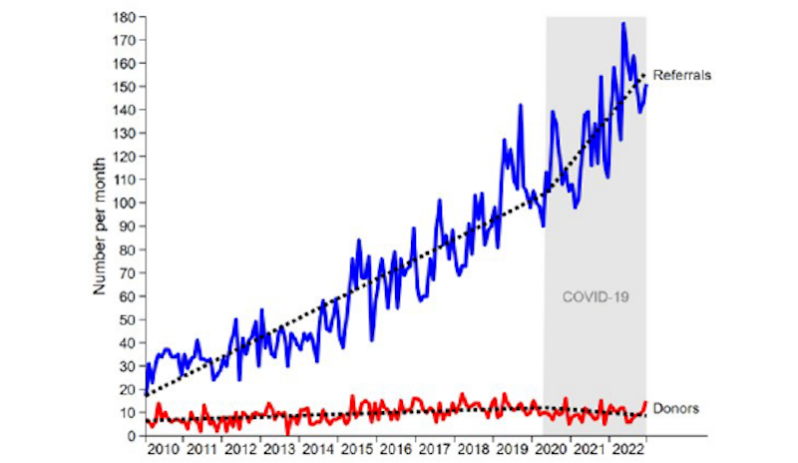
Number of deceased organ donor referrals who proceeded (actual donors) versus the number referred to the NSW OTDS for consideration for donation (potential donors) in NSW, 2010-2024. NSW: New South Wales; OTDS: Organ and Tissue Donation Service.

Improvements in donation rates in Australia have occurred unevenly. NSW has the greatest number of donors each year nationally. However, donation rates have not improved as much as in other states when standardized for population size (Figure 2) [13]. Furthermore, despite significant increases in potential donors referred to the NSW OTDS, our prior work has shown that the proportion of actual donors has increased at a much slower rate (Figure 3) [13]. Nationally, less than 42% of potential donors proceeded to actual donations in 2020 [5]. In NSW, only 22% of potential donors proceed to actual donation [14]. Improving donation in NSW, Australia’s most populous state, would greatly impact national donation levels and outcomes for people on the kidney transplant waitlist.

Preventing the transmission of donor-derived infectious diseases or cancer (biovigilance) is a central concern in transplant programs. Donor medical suitability assessment is based on the perception of the biovigilance risk a donor poses. Evidence of an infectious disease, current or historic malignancy, and behaviors that increase the risk of blood-borne viruses is often obtained indirectly from hospital records, primary care records, family members, or friends. There is varied access to past medical records and limited possibilities to confirm risk within the donor assessment time frame for deceased donors. In this context, the actual risk of disease transmission may be unconfirmed, and transplant clinicians make recommendations and decisions under conditions of uncertainty. Donation decisions for deceased donor organs are time sensitive and may vary among clinicians. We have demonstrated previously that even when clinicians correctly interpret clinical information related to biovigilance, their recommendations of donor suitability may be inconsistent [15]. Furthermore, many of the surveyed clinicians were found to be risk averse when making donor decisions for their patients, even when the blood-borne virus risk of transmission from donor to potential recipient was <1:10,000 [15]. Further opportunities exist to increase the deceased donor pool among those currently excluded due to biovigilance concerns.

Our prior work has identified several potential missed opportunities for deceased organ donation in NSW. Of over 3000 potential donors referred between 2010 and 2015, approximately 10% had increased risk behaviors for blood-borne viruses (hepatitis B, hepatitis C, and HIV), but the majority were deemed not medically suitable for organ donation without specific testing to confirm or refute virus exposure [16]. Those potential donors who were tested showed a <1 in 100,000 risk of having the disease. Similarly, 29% of potential donors who were deemed not medically suitable due to their perceived risk of cancer transmission had a tumor with a low risk of transmission when verified by cancer registry records, and the use of their organs for transplantation would have been within clinical transplantation guidelines [17]. The inclusion of potential deceased organ donors with a low risk of disease transmission but currently foregone as donors may increase the donor pool and opportunities for transplantation. However, the potential impact of such practice changes and the downstream impact on the kidney transplant waitlist, patient outcomes, and health care costs are unknown.

In this study, we aim to investigate whether increasing kidney donor acceptance can improve access to kidney transplants for people with kidney failure in 4 related projects:

- Kidney transplant waitlist dynamics: Using binational individual-level data, we will describe and evaluate factors impacting the current patient journey on the kidney transplant waitlist, including all clinical transitions: suspension and reactivation, death before transplant, and transplantation.
- Impact of donor decline for patients on the kidney transplant waitlist: Using our linked data as the cohort study platform, we will use flexible parametric survival models to quantify the time from offer decline to deceased donor transplantation and the impact of the intersectional disadvantage on the waiting time after decline for patients on the kidney transplant waitlist.
- Deceased donor profiling: Using our linked data as the cohort study platform, we will describe donor referral risk profiles and determine any potential donor gains that could be made through better access to donor information at the time of decision-making, more accurate estimation of the absolute biovigilance risk, and varying of the acceptable biovigilance risk thresholds for accepting donors.
- Economic modeling of increased donor acceptance: Using health economic models (cohort- and individual patient-level simulations), we will quantify the impact of varying donor referral decisions on health care costs, health benefits (quality-adjusted survival), and efficiency measures (time on the waitlist and time to a kidney transplant).

## Methods

### Study Design

The MODUS (Maximizing Organ Donor Utility Systemwide) study will use a variety of existing data sources and methods to investigate (1) kidney transplant waitlist dynamics, (2) the impact of declining a first donor offer on patients on the kidney transplant waitlist, (3) deceased organ donor biovigilance risk profiles, and (4) the health care costs and benefits of increased deceased organ donor acceptance. All analyses will be performed using STATA, R and TreeAGE software.

#### Kidney Transplant Waitlist Dynamics

We will include all people who entered the kidney transplant waitlist for their first transplant between July 1, 2010, and December 31, 2019, in NSW from the Australian and New Zealand Dialysis and Transplantation Registry (ANZDATA) and OrganMatch. ANZDATA is a collaborative disease registry collecting clinical and treatment information about people undergoing dialysis or transplants for kidney failure from all treatment centers in Australia and New Zealand, updated annually since 1977. OrganMatch is a clinical transplant system that facilitates compatibility matching of recipients and donors for organ transplantation and maintains the kidney transplant waiting list. We will exclude those who had multiorgan transplants (eg, kidney + pancreas). Our study follow-up will be from waitlist entry until death, or December 31, 2019.

We will build a multistate model of the kidney transplant waitlist and use this to describe waitlist dynamics and evaluate all clinical transitions after entering the waitlist. Clinical states will include active on the kidney transplant waitlist, temporary or permanent suspension, kidney transplant (deceased donor, living donor, and paired kidney exchange donor), and death before transplant. We will exclude people who were already active on the waitlist (ie, prevalent cases). Posttransplant clinical states will include graft failure, return to dialysis, and re-entry into the waitlist for subsequent transplant. We will evaluate patient factors associated with health state transitions after waitlist entry. Patient factors selected a priori include sex, age at waitlist entry, ethnicity, comorbidity burden, and cause of kidney failure. Specifically, we will estimate transition intensity hazard ratios to indicate whether a transition is more or less likely to occur based on patient factors. Transitions of interest include waitlist entry to suspension, suspension to return to waitlist, suspension to death before transplant, and waitlist entry to transplant.

#### Impact of Donor Decline for Patients on the Kidney Transplant Waitlist

Using the ANZDATA and OrganMatch linked data detailed before, we will investigate patients who entered the kidney transplant waiting list in NSW from 2010 to 2020. We will use records of ranked offers for every actual donor against potential recipients. Using records of which donor-recipient pairs were accepted, we will determine which donor-recipient pairs were offered but declined by transplant teams. We will use logistic regression to estimate the odds ratios of decline of the first donor-recipient paired offer by prespecified clinically relevant recipient characteristics, including blood group, sex, age, ethnicity, immunological sensitization by panel reactive antibody (PRA), and comorbidities. Subsequent outcomes after the decline of the first offer will be examined, including the time to the next offer, the time to the next better offer (measured as the next donor offer with improved an kidney donor profile index [KDPI]), the number of offers to transplant if transplanted, the rate and duration of the time spent suspended from the list, receiving a transplant (deceased or living donor), and death. The time from decline to transplantation will be examined, by patient characteristics, using Kaplan-Meier curves and the restricted mean survival time. We will use flexible parametric survival models to examine the time from decline to deceased donor transplantation for patients with different characteristics and to make adjusted predictions on the time from decline to transplantation, evaluating the impact of the intersectional disadvantage for patients with different sociodemographic and clinical characteristics. Finally, we will assess whether the decline of the offer leads to the subsequent transplant of a better-quality kidney; KDPIs of declined kidneys will be compared to those of accepted kidneys using the sign test for matched pairs.

#### Deceased Donor Profiling

To profile donors, we will use an established dataset that contains outcome data for all candidates for deceased organ donors referred to the NSW OTDS from January 1, 2010, to December 31, 2020, including those who proceeded to donation (actual donors), were deemed suitable for donation but never proceeded (intended donors), and were foregone for medical unsuitability (potential donors foregone) [14].

We will investigate potential donors foregone who were deemed not medically suitable in NSW. Specifically, we will summarize prespecified demographic and clinical characteristics, including age, sex, blood group, comorbidities, and the calculated Australian KDPI, of all consented potential donors and compare those declared medically unsuitable for donation with those who did proceed to donation. We will summarize the given reasons these potential donors did not proceed to donate. Lastly, we will determine the proportion of potential donors foregone due to concerns around medical suitability but who were also of better or the same quality as those who proceeded to donate, overall and by calendar year.

#### Economic Evaluation of Increased Donor Acceptance

To quantify the impact of varying donor acceptance on health care costs, benefits, and efficiency, we will use individual-level simulation-based Markov models to compare the cost-effectiveness of the shift in clinical practice that encourages increased use of potential donors with specific biovigilance concerns but who are within current guidelines (eg, history of cancer or increased risk of infectious diseases, including blood-borne viruses) in the Australian health care setting. We will adopt a payer (Australian government) perspective. Biovigilance risk thresholds for acceptance of deceased organ donors will follow prevailing clinical guidelines [6]. Health states included in our analysis include being active on the kidney transplant waitlist, being suspended from the waitlist, having a functioning kidney transplant, transplant failure and return to dialysis, death from kidney failure, and death from other causes, as well as health states specific to cancer or blood-borne viruses.

The transition of kidney patients through different health states over a lifetime time horizon will be simulated based on transition probabilities over 3-month time periods (Markov cycles) derived from the published literature. Health outcomes will be measured in terms of life-years gained and quality-adjusted life years (QALYs) gained, which incorporates both the length and the quality of life (scale 0-1, where 0=death and 1=full health), based on the published literature. Costs will be measured in Australian dollars using data obtained from the Medicare Benefits Schedule, the Pharmaceutical Benefits Scheme, and relevant unit pricing based on Australian-Refined Diagnosis Related Group codes and the Independent Hospital Pricing Authority Net Efficient Price. A discount factor of 5% per year will be applied to costs and health outcomes, as per Australian government recommendations [18] of 2016 and Medical Services Advisory Committee recommendations of 2021 [19].

The cost-effectiveness of the shift in clinical practice to accept deceased donor organs with varying biovigilance risks, the risk of cancer, and the risk of blood-borne virus transmission aligned with current guidelines will be compared with the current practice, where these donors are typically declined. Cost-effectiveness will be measured as a ratio of incremental costs to incremental QALYs expressed as an incremental cost-effectiveness ratio (ICER) [20] or net monetary benefit. A willingness-to-pay threshold will be determined based on estimates from the literature per QALY gained and used to interpret the ICER. A strategy will be interpreted as cost-effective if the ICER is less than the willingness-to-pay threshold per QALY gained. Sensitivity analyses will be conducted using varying willingness-to-pay thresholds.

We will use individual-level simulation models to overcome some of the limitations of cohort-based Markov models, as well as to incorporate prespecified individual recipient and donor characteristics [21,22]. Recipient-level characteristics, such as age, sex, blood group, number of previous kidney transplants, dialysis vintage, and comorbidities, and donor characteristics, such as age, sex, donor type (donation by brain or circulatory death), and Australian KDPIs, will be used. We plan to undertake distributional cost-effectiveness analysis, a framework to incorporate health inequality impacts into the cost-effectiveness analysis comparing shifts in the clinical practice to accept deceased organ donors with an increased risk of cancer and blood-borne virus transmission with the current practice where these donors are typically declined [23]. The model structure and analyses will follow best-practice modeling guidelines from the International Society for Pharmacoeconomics and Outcomes Research [24]. Comprehensive deterministic and probabilistic sensitivity analyses will be conducted to account for all parameter uncertainties in the models.

### Consumer Engagement

The MODUS study has been designed with input from peak consumer organizations and key stakeholders in the delivery of Australia’s organ donation and transplantation service. Our research partners have been integral to the development of this study protocol; the NSW Ministry of Health has underwritten the Biovigilance Public Health Register and will contribute to outcome development, and the NSW OTDS will contribute expertise and help fidelity through close relationships with medical suitability advisors and transplanting hospitals; Kidney Health Australia has provided an ongoing vital consumer liaison. Increasing opportunities for transplantation are a priority for people with kidney disease. Our consumer liaisons will advise on project development, interpretation of results, and dissemination strategies for research findings.

### Ethical Considerations

This study was approved by the University of Sydney Human Research Ethics Committee (project number 2020/828). ANZDATA obtained approval for the collection and secondary analysis of data of all patients included in the study by the Royal Adelaide Hospital Research Ethics Committee (approval number HREC/17/RAH/408R20170927). A waiver of consent was provided for data obtained from the NSW OTDS, as all participants were deceased. All data provided to research staff were deidentified to maintain patient privacy and confidentiality.

## Results

The MODUS study was funded by the National Health and Medical Research Council (APP1171364) in December 2018. We received the linked health data from the Centre for Health Records Data Linkage in May 2023. We received data for 20,818 individuals, 3812 patients ever entered on the kidney transplant waitlist, and 8707 potential donors referred for consideration for deceased organ donation. Since October 2024, data analysis is ongoing for the 4 MODUS study aims. Study findings will be disseminated between 2025 and 2026 as the analysis for each project is completed.

## Discussion

### Summary

The MODUS study hypothesizes that increasing the acceptance of deceased kidney donors who are currently declined due to biovigilance concerns will lead to significant improvements in health outcomes for people with kidney failure. Specifically, the study anticipates that increased donor acceptance will increase the number of kidney transplants, reduce kidney transplant waiting times, and improve overall patient survival and quality of life. In addition, we hypothesize that increasing donor acceptance will also prove to both benefit patients with kidney failure and be cost-effective.

Our work is expected to generate critical evidence to support policy and practice changes: detailed insights into the factors influencing patient transitions on the kidney transplant waitlist, including suspension, reactivation, and transplantation; quantification of the time from donor offer decline to transplantation and the impact of the intersectional disadvantage on waiting times; identification of potential donor gains through improved access to donor information and more accurate biovigilance risk assessments; and evidence of the cost-effectiveness of increasing donor acceptance, measured in terms of health care costs, QALYs, and efficiency metrics.

### Comparison With Prior Work

Previous research has primarily focused on factors affecting access to the kidney transplant waitlist and posttransplant outcomes. However, the MODUS study will extend beyond this scope of work to uniquely address the underexplored area of waitlist dynamics and the impact of deceased donor offer declines. By integrating binational data and advanced statistical models, this study will provide a comprehensive analysis that extends beyond the scope of prior work. Additionally, the economic modeling component will offer a novel perspective on the cost-effectiveness of policy changes in organ donation.

Organ donation systems vary significantly across countries, impacting donation rates and transplant outcomes. For instance, Spain’s opt-out system has led to one of the highest organ donation rates worldwide, with 46 deceased donors per million population [25]. In contrast, countries such as the United States and Australia, which primarily use opt-in systems, have lower donation rates. The MODUS study’s findings could inform the potential benefits of adopting more inclusive donor acceptance criteria, similar to Spain’s approach, to increase donor pools and improve transplant outcomes.

### Strengths and Limitations

Our study will use linked and registry-based health service data from multiple sources to provide robust estimates of patient and health service outcomes to inform our health economic assessment of interventions that could increase deceased organ donor acceptance. Yet, most of these data were not collected for research purposes and so may not contain granular patient information that could provide further insights, such as reason for kidney transplant waitlist suspension. There may also have been changes in clinical practices and donor referral processes during our look-back period that could impact our analyses. However, deceased organ donor trends and transplant outcomes have remained relatively stable over time. In addition, there may be missing data where we would use a complete case approach or multiple imputation. Our study’s findings are highly relevant to the Australian context, but their applicability to other health care systems may be limited. The likelihood of physicians using the tools developed in the MODUS study will depend on several factors, including usability, feasibility, and user preferences. The study includes plans to assess these aspects during development to ensure successful implementation. Engaging with health care professionals through surveys and focus groups will help refine the tools and ensure they meet clinical needs. Training programs and integration with existing clinical workflows will also be essential to promote adoption.

### Dissemination Plan

The findings of the MODUS study will be disseminated through multiple channels to ensure a broad reach and impact. We will present results at national and international scientific conferences to engage with both academic and clinical communities. We will submit research manuscripts to peer-reviewed journals to contribute to the scientific literature, prioritizing open access journals. We will also share our findings with our listed partner organizations (the NSW OTDS) and external stakeholders (the Australian Organ Transplant Authority and Kidney Health Australia) to facilitate knowledge transfer and plan appropriate interventions for implementation.

### Conclusion

The MODUS study aims to provide compelling evidence that increasing the acceptance of deceased donor kidneys, currently declined due to biovigilance concerns, can significantly improve patient outcomes and be cost-effective. By addressing critical gaps in the understanding of kidney transplant waitlist dynamics and donor profiling, this study has the potential to inform policy and practice changes that enhance organ donation and transplantation processes, ultimately benefiting patients with kidney failure.

## Abbreviations

ANZDATA: Australian and New Zealand Dialysis and Transplantation Registry
ICER: incremental cost-effectiveness ratio
KDPI: kidney donor profile index
MODUS: Maximizing Organ Donor Utility Systemwide
NSW: New South Wales
OTDS: Organ and Tissue Donation Service
QALY: quality-adjusted life year

## Data Availability

The data that support the findings of this study are not publicly available due to their containing information that could compromise the privacy of research participants, but summary-level data may be available from the Collaborative Centre for Organ Donation Evidence (CODE), University of Sydney, upon reasonable request. The data collated for this study are under the management of the New South Wales Ministry of Health as a Public Health Register.
